# Brief communications: changes in inflammatory biomarkers and lipid profiles after switching to long-acting cabotegravir plus rilpivirine

**DOI:** 10.1186/s12981-023-00590-4

**Published:** 2024-01-03

**Authors:** Eisuke Adachi, Makoto Saito, Amato Otani, Michiko Koga, Hiroshi Yotsuyanagi

**Affiliations:** Department of Infectious Diseases and Applied Immunology, IMSUT Hospital of The Institute of Medical Science, The University of Tokyo, 4-6-1 Shirokanedai, Minato-ku, Tokyo, 108-8639 Japan

**Keywords:** Cabotegravir plus rilpivirine, Inflammatory biomarker, Lipid profile, HIV, Long-acting drug

## Abstract

We assessed whether the impact of cabotegravir plus rilpivirine on inflammation reduction differs from that of oral antiretrovirals, using real-world data. Inflammatory biomarkers and lipid profiles were followed from baseline to 8 months after switching. Seventy-eight participants were analyzed. The CD4/CD8 ratio and C-reactive protein did not change. There were transient decreases in CD8 and CD4 counts in the group that switched from the dolutegravir-based regimen, but not in the tenofovir alafenamide-based regimen group. High-density lipoprotein (HDL) cholesterol increased, resulting in a decrease in the total-cholesterol to HDL cholesterol ratio, whereas there was no significant change in low-density lipoprotein cholesterol.

**Supplementary Information:**

The online version contains supplementary material available at 10.1186/s12981-023-00590-4.

## Introduction

Chronic inflammation once elevated by persistent HIV infection does not return to the levels seen in non-HIV-infected individuals, even on effective antiretroviral therapy (ART) [1, 2]. Persistent HIV-related inflammation is known to contribute to an increased risk of non-AIDS-related complications such as cardiovascular disease. Continued viral replication in the HIV reservoir, overlapping infections, and bacterial translocation are thought to contribute to chronic inflammation [3, 4]. Inflammatory biomarkers that have been reported to be associated with HIV infection include C-reactive protein (CRP), interleukin-6 (IL-6), monocyte and macrophage activation biomarkers (e.g., soluble CD14 [sCD14] and soluble CD163), T cell activation biomarkers (e.g., CD4/CD8 ratio), atherosclerosis and hypercoagulation (e.g., D-dimer) [3, 5, 6]. The CD4/CD8 ratio is associated not only with chronic inflammatory diseases but also with immunosenescence [6], which reduces the immune response to novel antigens [7, 8].

Inflammatory biomarkers have been used as endpoints in clinical trials, particularly those comparing two-drug regimens to three-drug regimens [9]. In a real-world data from Japan, we showed that inflammatory biomarkers did not change after switching to dolutegravir/lamivudine (DTG/3TC), but there was a mild improvement in lipid profiles [10].

Long-acting cabotegravir plus rilpivirine (CAB plus RPV) has been reported to have higher satisfaction than oral ART [11–13]. On the other hand, CAB plus RPV is a treatment that regularly invades the gluteus and the administration of drugs by injection has also been noted to cause inflammation of the gluteus medius with fever [12, 14, 15].

No real-world data on inflammatory biomarkers or lipid profiles after switching to CAB plus RPV in Asians have been reported to date. Herein, we compared CRP, CD4/CD8 ratio and lipid profiles measured in daily clinical practice before and after switching to CAB plus RPV.

## Methods and populations

In this retrospective cohort study, we reviewed routinely collected clinical records of people with HIV (PWH) who received injectable CAB plus RPV at IMSUT hospital of Institute of Medical Science, The University of Tokyo (an HIV/AIDS referral hospital in urban Tokyo), between June 2022 and May 2023. Only PWH who had been on injectable CAB plus RPV for at least 5 months were included. All participants had been on oral CAB plus RPV (oral lead-in [OLI]) for at least 1 month. At each visit from the start of OLI until 7 months after injectable CAB plus RPV administration, i.e., approximately 8 months after the start of OLI, data on inflammatory biomarkers (i.e., CRP, CD4/CD8 ratio), lipid profiles (i.e., High-density lipoprotein cholesterol (HDL-c), Low-density lipoprotein cholesterol (LDL-c), Total cholesterol [T-chol] /HDL-c) were collected. Only test results conducted at regular HIV outpatient visits were included, and the results at the irregular visits for other complaints (e.g. acute infectious diseases) and at the follow-ups for complications were excluded from the analysis. In addition, in participants who added dyslipidemia medications after switching to CAB plus RPV, the data after the start of those drugs were excluded from the analysis. Virological failure was defined as HIV-RNA ≥ 200 copies/mL at the subsequent test when HIV-RNA was initially above 50 copies/mL. To determine the impact of tenofovir alafenamide (TAF) on lipid and inflammatory biomarkers, participants were grouped depending on the regimen prior to switching to CAB plus RPV: TAF-based regimen group and DTG-based regimen group. TAF-based regimen group included bictegravir/emtricitabine/tenofovir alafenamide(B/F/TAF), DTG plus F/TAF, raltegravir plus F/TAF and RPV plus F/TAF, but not regimens that contain protease inhibitor or boosters. The DTG-based regimen included DTG/3TC and abacavir/lamivudine/DTG.

Repeated measures analysis of variance (ANOVA) was used to assess whether inflammation biomarkers and lipid profiles changed statistically at different time points. We used the Wilcoxon signed-rank test to evaluate the difference between biomarkers before and after switching to CAB plus RPV.

## Results

A total of 78 individuals were eligible for the study. There were 42 individuals in the TAF-based regimen group and 32 in the DTG-based regimen group, with no statistical difference between these two groups in their respective background characteristics other than CD8 cell counts (p = 0.015, Additional file 1). There were no virological failures; only 2 individuals had HIV-RNA > 50 copies/mL at month 0, and 2 participants at month 1. Approximately 80% of the baseline cases were undetectable (i.e., target not detected) (see Additional file 1).

**Fig. 1:**
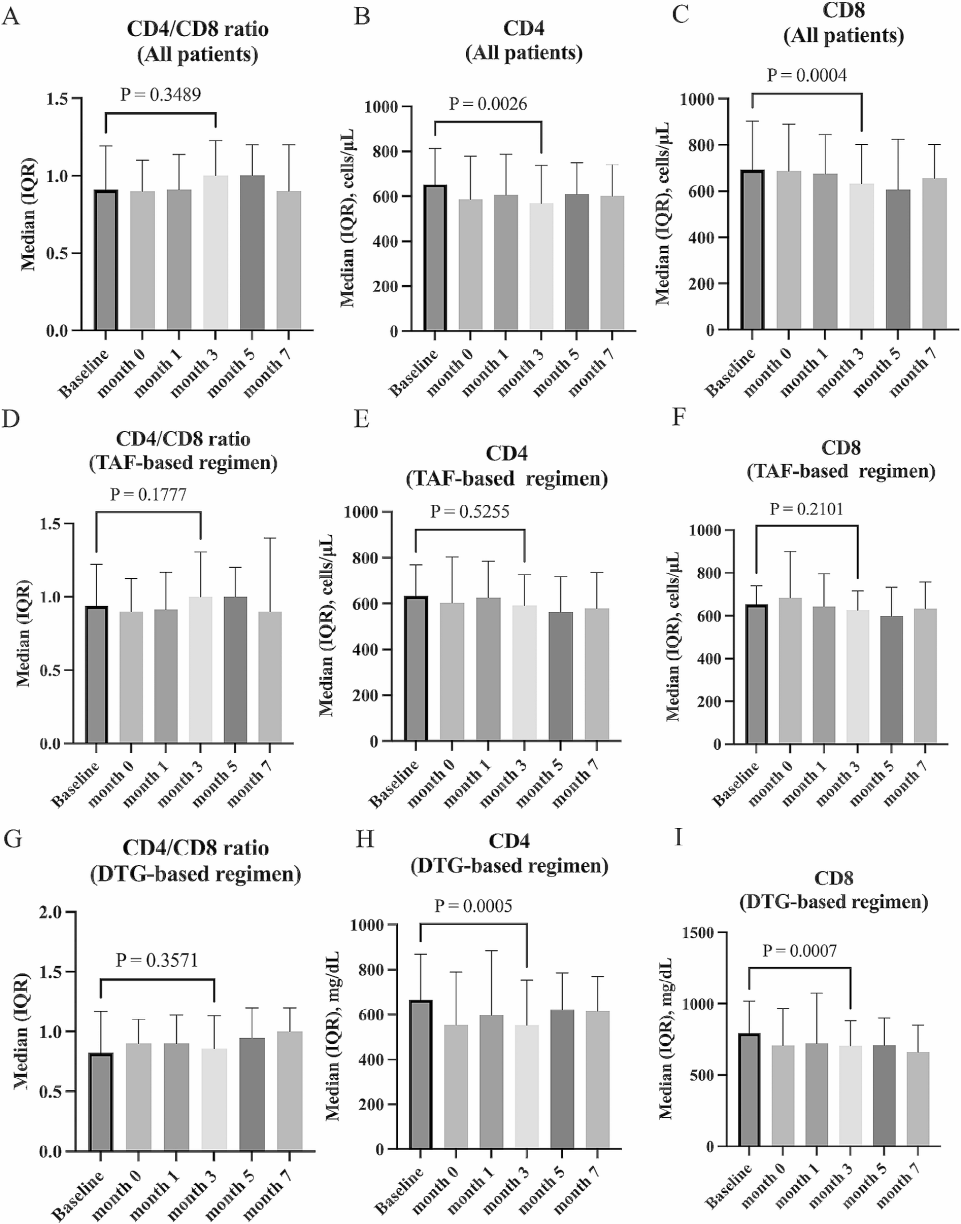
Changes in CD4/CD8 ratio after switching to cabotegravir plus rilpivirine. Changes in CD4/CD8 ratio (**A**), CD4 counts (**B**), and CD8 counts (**C**) before and after the switch to cabotegravir plus rilpivirine in all participants are shown. The analysis for each antiretroviral therapy regimen prior to switching shows changes in CD4/CD8 ratio (**D**), CD4 counts (**E**), and CD8 counts (**F**) in the TAF-based regimen group, and changes in CD4/CD8 ratio (**G**), CD4 counts (**H**), and CD8 counts (**I**) in the DTG-based regimen group. Baseline, at the start of oral-lead-in; month 0, at the start of injectable cabotegravir plus rilpivirine. The p-values in the graphs are calculated by the Wilcoxon signed-rank test comparing the values at baseline and at month 3. TAF, tenofovir alafenamide; DTG, dolutegravir

The change in inflammation biomarkers after switching to CAB plus RPV was examined using repeated measures ANOVA. When all participants regardless of pre-switching regimens were combined, no significant change in CRP (p = 0.52) was found (see Additional file 2). There was no significant change in CRP in either the TAF-based regimen group (p = 0.35) or DTG-based regimen group (p = 0.68). In all participants combined, no significant change in CD4/CD8 ratio (p = 0.44) was found. The p-values in Fig. 1 were results of the Wilcoxon signed-rank test. CD8 counts decreased at month 3 (median of difference − 57 cells/µL p = 0.0004, Fig. 1C). CD4 counts also decreased at month 3 (median of difference − 44 cells/µL, p = 0.0026, Fig. 1B) and at month 7 (median of difference − 33 cells/µL, p = 0.043). In the TAF-based regimen group, there was no significant change in either CD4 counts (p = 0.53) or CD8 counts (p = 0.20) between different time points using repeated measures ANOVA. On the other hand, in the DTG-based regimen group, the CD4/CD8 ratio (p = 0.056) was not significantly different, but CD4 counts (p = 0.0018) and CD8 counts (p = 0.0016) were significantly decreased. In the DTG-based regimen group, theses biomarkers at baseline OLI initiation and at month 3 were compared by Wilcoxon signed-rank test. CD4 counts (median of difference − 76 cells/µL, p = 0.0005, Fig. 1H) and CD8 counts (median of difference − 94 cells/µL, p = 0.0007, Fig. 1I) were decreased significantly.

**Fig. 2:**
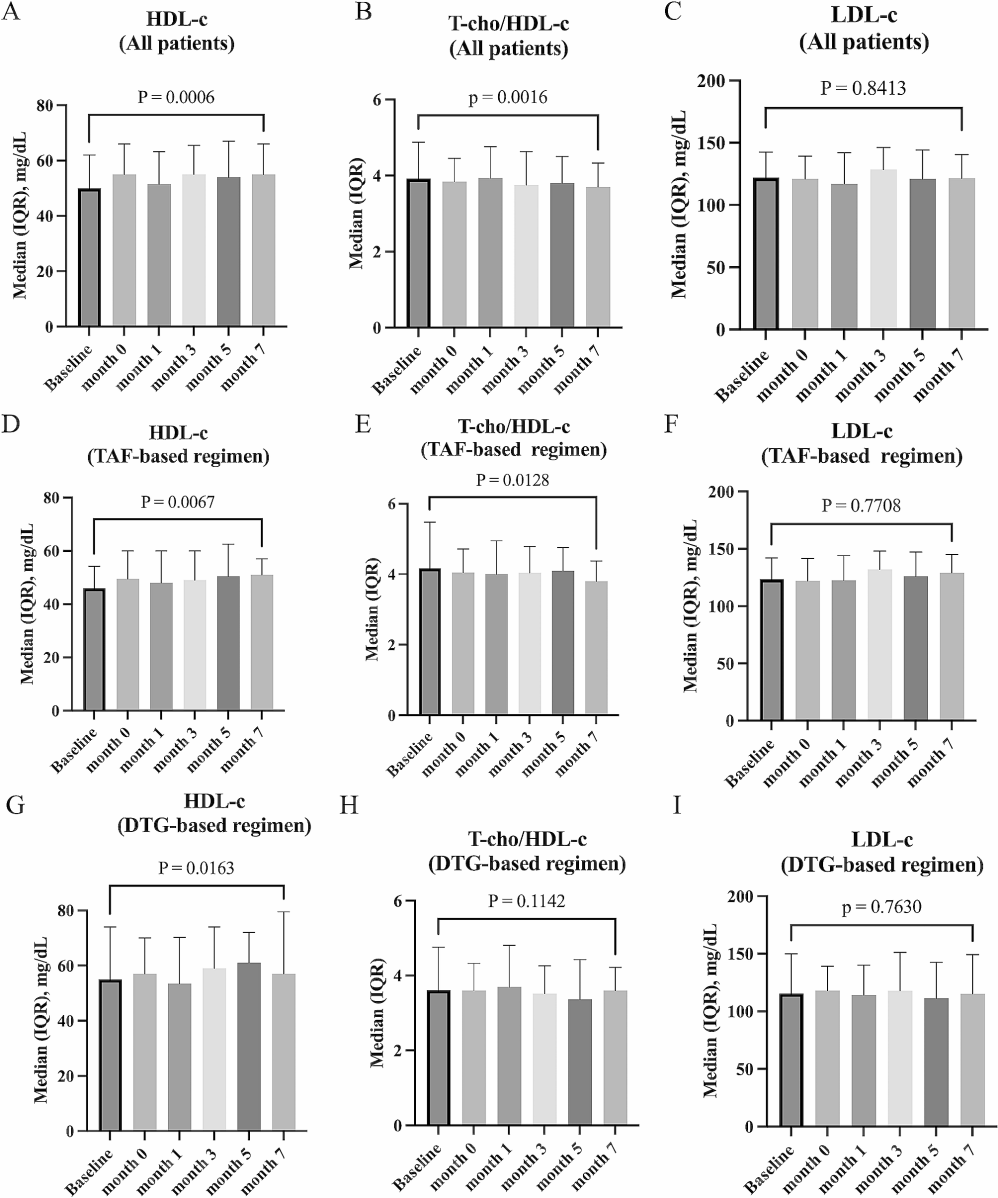
Changes in lipid profiles after switching to cabotegravir plus rilpivirine. Changes in HDL-c (**A**), T-chol/HDL-c (**B**), and LDL-c (**C**) during the switch to cabotegravir plus rilpivirine in all participants are shown. The analysis for each antiretroviral therapy regimen prior to switching shows changes in HDL-c (**D**), T-chol/HDL-c (**E**), and LDL-c (**F**) in the TAF-based regimen group, and changes in HDL-c (**G**), T-chol/HDL-c (**H**), and LDL-c (**I**) in the DTG-based regimen group. Baseline, at the start of oral-lead-in; month 0, at the start of injectable cabotegravir plus rilpivirine. HDL-c, high-density lipoprotein-cholesterol; T-chol, total cholesterol; LDL-c, low-density lipoprotein-cholesterol. The p-values in the graphs are calculated by the Wilcoxon signed-rank test comparing the values at baseline and at month 7. TAF, tenofovir alafenamide; DTG, dolutegravir

The change in lipid profiles after switching to CAB plus RPV was examined using repeated measures ANOVA. Including all participants regardless of pre-switching regimens, after switching to CAB plus RPV, there was a significant increase in HDL-c (p = 0.0001), a significant decrease in T-chol/HDL-c (p = 0.0013), whereas LDL-c did not change after switching to CAB plus RPV (p = 0.52). Similar changes were observed in the TAF-based and DTG-based regimen groups, and there was no difference in the trend of lipid profile changes regardless of the regimen prior to switching. The extent to which the lipid profile had actually changed was shown in Fig. 2.

## Discussions

In this study, there were no significant changes in inflammatory biomarkers (i.e., CRP and CD4/CD8 ratio) regardless of the regimen prior to switching to CAB plus RPV. A temporary decrease in CD4 and CD8 counts was observed after switching to CAB plus RPV in the DTG-based regimen group. The decrease in CD8 counts may lead to an improvement in T cell-mediated chronic inflammation [16, 17]. A decrease in CD4 counts could be a serious problem related to cellular immunodeficiency, however the CD4 counts appeared to improve in month 7. Furthermore, the simultaneous decrease in CD4 and CD8 may be associated with acute inflammation after the administration of injectables, but the association is unclear because CRP levels did not change. No change in CD4 or CD8 counts was observed in the TAF-based regimen group. The sole significant disparity in baseline background factors between the two groups is the higher CD8 counts in the DTG-based regimen group compared to the TAF-based regimen group (see Additional file 1).

Lipid profiles could be associated with chronic inflammation. In this study, after switching to CAB plus RPV, T-chol/HDL-c improved significantly, while LDL-c remained unchanged. Because of the lipid-lowering effect of tenofovir disoproxil fumarate (TDF) [18], it is often observed in clinical practice that switching from TDF to TAF raises lipids, but the effect of TAF on lipids has not been clearly understood so far. In this study, there was no effect of TAF on lipid profiles. By contrast, in our previous study, we examined the change in lipid profile by switching from B/F/TAF or F/TAF plus DTG to DTG/3TC and found an increase in HDL-c and a decrease in T-chol/HDL-c, suggesting that TAF may affect the lipid profile [10]. Further data are required to examine the causes of the discrepancy in these results. It may also be difficult to conclude whether switching to CAB plus RPV will improve the prognosis of PWH even if HDL-c increases compared with the use of oral antiretroviral drug, because an increase in HDL-c alone without a decrease in LDL-c may not be associated with a reduced the risk of the development of chronic inflammatory diseases [19].

This study has several limitations. First, the observation period is relatively short. However, concerning viremia and ISR, which can significantly impact inflammation, over 85% of virological failures in three pivotal clinical trials occurred within 8 months, aligning with the observation period in this study [11, 12, 20], and ISRs were most common after the first dose following the switch to CAB plus RPV, then decreased, and the frequency remained consistent after the third dose [12]. Second, the data of lipid profiles don’t entirely eliminate the impact of their diet on those levels. Finally, we did not evaluate inflammation-related biomarkers such as IL-6, sCD14 or D-dimer, which have been reported to be associated with non-AIDS-related diseases in previous studies [1, 6]. This study was a secondary analysis of routinely collected clinical data, and IL-6, sCD14 and D-dimer were not routinely monitored; previous studies that evaluated inflammatory biomarkers using real-world data also evaluated CRP and CD4/CD8 ratio, which were measured in routine clinical practice [9].

In conclusion, there was no significant change in inflammatory biomarkers, but there was an improvement in T-chol/HDL-c after switching from oral regimen to injectable CAB plus RPV in daily clinical practice. The efficacy and safety of injectable CAB plus RPV are not inferior to conventional oral ART when chronic inflammation is the endpoint.

### Electronic supplementary material

Below is the link to the electronic supplementary material.

**Additional file 1:** **Table.** Demographic characteristics at baseline

**Additional file 2:** **Figure.** Changes in C-reactive protein after switching to cabotegravir plus rilpivirine. The changes in C-reactive protein after switching to cabotegravir plus rilpivirine in all participants (A), TAF-based regimen group (B), and DTG-based regimen group (C), respectively, are shown. Baseline, at the start of oral-lead-in; month 0, at the start of injectable cabotegravir plus rilpivirine. TAF, tenofovir alafenamide; DTG, dolutegravir

## Data Availability

The data that support the findings of this study are available on request from the corresponding author. The data are not publicly available due to privacy or ethical restrictions.

## List of abbreviations

ART: antiretroviral therapy
CRP: C-reactive protein
IL-6: interleukin-6
sCD14: soluble CD14
DTG/3TC: dolutegravir/lamivudine
TAF: tenofovir alafenamide
CAB plus RPV: Long-acting Cabotegravir plus rilpivirine
PWH: people with HIV
OLI: oral lead-in
HDL-c: high density lipoprotein cholesterol
LDL-c: Low-density lipoprotein cholesterol
T-chol: Total cholesterol
B/F/TAF: bictegravir/emtricitabine/tenofovir alafenamide
ANOVA: analysis of variance
TDF: tenofovir disoproxil fumarate
