# Supplementary material for: Brief communications: changes in inflammatory biomarkers and lipid profiles after switching to long-acting cabotegravir plus rilpivirine
Source: AIDS Res Ther. 2024 Jan 3;21:1. doi: 10.1186/s12981-023-00590-4 (PMC10765943; doi:10.1186/s12981-023-00590-4)
Supplement: Supplementary file 1 — Additional file 1: Table. Demographic characteristics at baseline [file 12981_2023_590_MOESM1_ESM.pdf]

Additional file 1

Table Demographic characteristics at baseline

ART regimen before switching		All ART regimens	TAF-based Regimen	DTG-based Regimen	P value
n		78	42	32	
Age, years		48 (26-64)	47 (26-64)	50 (28-64)	0.16*
Gender	Male	76	41	31	0.999†
	Female	2	1	1	
Race	Asians	74	40	31	0.999†
	Other races‡	4	2	1	
Current smoker, n (%)		16 (21)	8 (19)	8 (25)	0.78†
Alcohol consumption, n (%)		39(50)	24 (57)	13 (41)	0.24†
CD4 cell count, cells/μL		655 (200-1210)	634 (200-1135)	656 (256-1210)	0.29*
CD8 cell count, cells/μL		694 (241-1874)	654 (241-1440)	795 (433-1874)	0.015*
CD4/CD8 ratio		0.91 (0.21-2.88)	0.94 (0.44-2.88)	0.82 (0.21-2.09)	0.32*
HIV-RNA < 50 copies/mL, n (%)		78 (100)	42 (100)	32 (100)	0.999†
HIV-RNA undetectable, n (%)		63 (80.8)	32 (76)	26 (81)	0.78†
Body mass index, kg/m²		24.2 (17.8-36.8)	24.2 (17.8-36.8)	24.0 (19.2-32.3)	0.51*
Time since diagnosis of HIV infection, years		11.8 (0.83–33.4)	12.2 (1.83–33.4)	11.2 (0.83–27)	0.85*
ART duration, years		10.3 (0.63-29.7)	10.04 (1.58-29.7)	10.75 (0.63-27)	0.64*
Complications, n (%)	Hypertension	14 (18)	6 (14)	8 (25)	0.37†
	Diabetes	8 (10)	5 (12)	3(9.4)	0.999†
	Dyslipidemia	21 (27)	11 (26)	7 (22)	0.78†
HIV-1 subtype§	B	69	36	29	0.999†
	Other subtypes§	7	4	3	
ART regimen before switch, n	DTG/3TC	30	0	30	NA
	B/F/TAF	37	37	0	
	Other regimens	11	5	2	

Data are n or median (range). P values were calculated comparing the TAF-based regimen group with the DTG-based regimen group.* Mann-Whitney U test, † Fisher's exact test , ‡ Southeast Asian 1, Latin American, and 2 Caucasian 1. § The other subtypes were AG 3, AE 1, D 2, and G 1. No data for 2 individuals. ART, antiretroviral therapy; TAF, emtricitabine/tenofovir alafenamide; DTG/3TC, dolutegravir plus lamivudine; B/F/TAF, bictegravir/emtricitabine/tenofovir alafenamide.
