# Supplementary material for: Brief communications: changes in inflammatory biomarkers and lipid profiles after switching to long-acting cabotegravir plus rilpivirine
Source: AIDS Res Ther. 2024 Jan 3;21:1. doi: 10.1186/s12981-023-00590-4 (PMC10765943; doi:10.1186/s12981-023-00590-4)
Supplement: Supplementary file 2 — Additional file 2: Figure. Changes in C-reactive protein after switching to cabotegravir plus rilpivirine. The changes in C-reactive protein after switching to cabotegravir plus rilpivirine in all participants (A), TAF-based regimen group (B), and DTG-based regimen group (C), respectively, are shown. Baseline, at the start of oral-lead-in; month 0, at the start of injectable cabotegravir plus rilpivirine. TAF, tenofovir alafenamide; DTG, dolutegravir [file 12981_2023_590_MOESM2_ESM.pdf]

Additional file 2

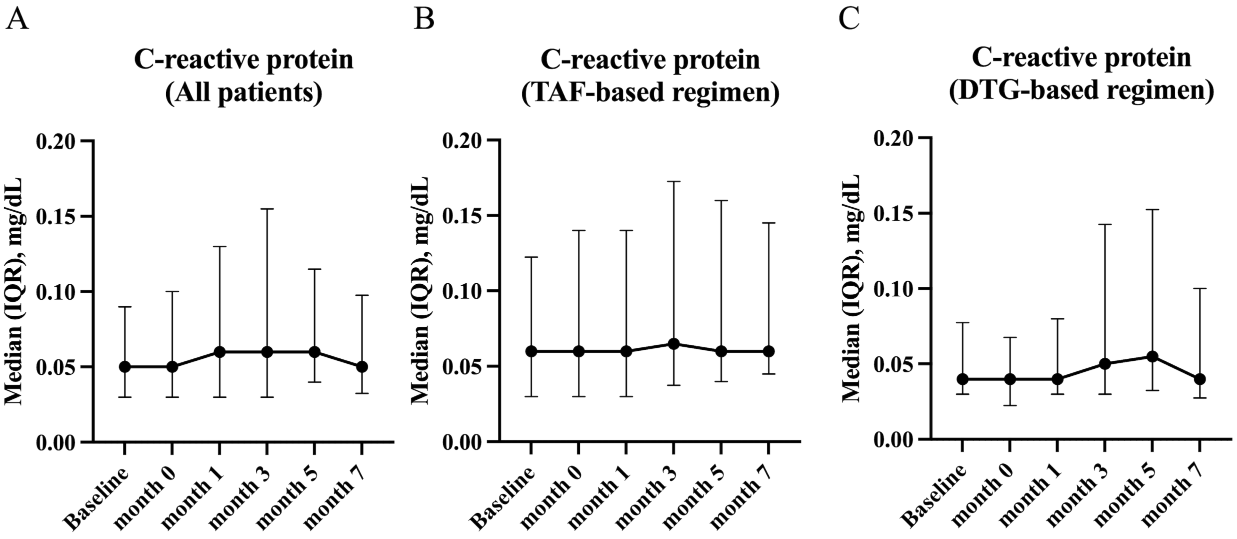

Figure. Changes in C-reactive protein after switching to cabotegravir plus rilpivirine.
The changes in C-reactive protein after switching to cabotegravir plus rilpivirine in all patients (A), TAF-based regimen group (B), and DTG-based regimen group (C), respectively, are shown. Baseline, at the start of oral-lead-in; month 0, at the start of injectable cabotegravir plus rilpivirine. TAF, tenofovir alafenamide; DTG, dolutegravir
